# Molecular design for recombinant adeno-associated virus (rAAV) vector production

**DOI:** 10.1007/s00253-017-8670-1

**Published:** 2017-12-04

**Authors:** Juan Jose Aponte-Ubillus, Daniel Barajas, Joseph Peltier, Cameron Bardliving, Parviz Shamlou, Daniel Gold

**Affiliations:** 1 Biomarin Pharmaceutical Inc., 105 Digital drive, Novato, CA 94949 USA; 2 Keck Graduate Institute of Applied Life Sciences, 535 Watson drive, Claremont, CA 91711 USA

**Keywords:** Adeno-associated virus, Gene therapy, Bioprocessing, Vector production

## Abstract

Recombinant adeno-associated virus (rAAV) vectors are increasingly popular tools for gene therapy applications. Their non-pathogenic status, low inflammatory potential, availability of viral serotypes with different tissue tropisms, and prospective long-lasting gene expression are important attributes that make rAAVs safe and efficient therapeutic options. Over the last three decades, several groups have engineered recombinant AAV-producing platforms, yielding high titers of transducing vector particles. Current specific productivity yields from different platforms range from 10^3^ to 10^5^ vector genomes (vg) per cell, and there is an ongoing effort to improve vector yields in order to satisfy high product demands required for clinical trials and future commercialization.

Crucial aspects of vector production include the molecular design of the rAAV-producing host cell line along with the design of AAV genes, promoters, and regulatory elements. Appropriately, configuring and balancing the expression of these elements not only contributes toward high productivity, it also improves process robustness and product quality. In this mini-review, the rational design of rAAV-producing expression systems is discussed, with special attention to molecular strategies that contribute to high-yielding, biomanufacturing-amenable rAAV production processes. Details on molecular optimization from four rAAV expression systems are covered: adenovirus, herpesvirus, and baculovirus complementation systems, as well as a recently explored yeast expression system.

## Introduction

Recombinant adeno-associated viruses (rAAV) have gained increasing attention in the viral gene therapy field. A safe clinical profile, availability of viral serotypes with different tissue tropisms, and potential long-term gene expression are main advantages of rAAV as viral vector. To date, more than 100 gene therapy clinical trials have been conducted, tackling a variety of diseases such as lipoprotein lipase (LPL) deficiency, cystic fibrosis, and hemophilic disorders (Carter 2005; Bryant et al. 2013; Gene therapy clinical trials website: http://www.abedia.com/wiley/).

Production of rAAV vectors started approximately 32 years ago, after different groups demonstrated formation of genetically modified AAV viral particles capable of infecting and transducing mammalian cells (Tratschin et al. 1984; Hermonat and Muczycska 1984). From a clinical perspective, these findings raised the possibility for developing a new type of therapeutic DNA vector. From a biotechnology and bioprocessing perspective, this new biologic represented a unique market opportunity, assuming that biotech manufacturing capabilities could meet quantity and quality requirements. Subsequently, significant effort has been put toward the design of simple and efficient processes for rAAV production.

Three expression systems are currently used for industrial vector production: adenovirus (AdV), herpesvirus (HSV), and baculovirus (BV) complementation systems. Despite promising advances, there are several challenges associated with the manufacturing process. Complementation systems bring inherent complexity to the production process because rAAV formation requires an intricate interplay between virus and host genetic elements. This phenomenon has a direct effect on process robustness and understanding, as subtle variations on the number of biological or chemical inputs used in upstream bioprocessing can affect productivity. This problem is clearly seen in transfection-based protocols, where lot-to-lot yield can vary drastically based on the number and concentration of plasmids used, amount of transfection agent used, cell viability, and mode of operation (Wright 2009; Huang et al. 2013; van der Loo and Wright 2016). Another concern during vector production refers to the generation of product-related impurities. Some components used during cell culture readily copurify with rAAV or are difficult to remove without damaging the rAAV product, making purification challenging. Among them, collateral packaging of non-AAV DNA has raised concerns because of its potential clinical implications (Wright 2014). Finally, vector yield is one of the most limiting factors for potential commercial supply. Current specific productivity yields from different platforms range from 10^3^ to 10^5^ vector genomes (vg) per cell, and there is an ongoing effort to improve vector yields to satisfy high product demands (Clark 2002; Ayuso et al. 2010). Overall, the best molecular constructs are the ones where the nature of AAV genes, promoters, and regulatory elements contribute to a simple, economic process that generates safe, high-quality vectors.

In this mini-review, the rational design of rAAV-producing expression systems is discussed, with special attention to molecular strategies that contribute to high-yielding, biomanufacturing-amenable rAAV production processes. In addition, in-depth details of a new microbial system for rAAV vector production based on *Saccharomyces cerevisiae* are provided. The molecular configuration proposed by different groups and their potential implications in vector production processes are discussed. Other aspects of gene therapy vector bioprocessing such as cell line and inoculum scalability, transfection optimization, and media optimization are covered elsewhere (Negrete and Kotin 2008; Kotin 2011; Thomas et al. 2009; Clement and Grieger 2016; Robert et al. 2017).

### rAAV vector biology

**Fig. 1:**
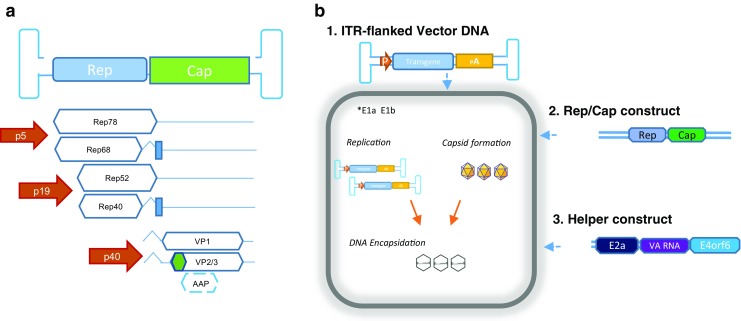
Adeno-associated virus (AAV) vector biology. Wild-type AAV genome (**a**) contains Rep and Cap genes. Rep encodes four regulatory proteins that play important roles in replication and encapsidation of viral DNA, and their expression is controlled by p5 and p19 promoters. Cap encodes three capsid proteins and assembly-activating protein (AAP), regulated by p40 promoter. In an AAV vector (**b**), the wild-type AAV Rep and Cap genes have been replaced with the transgene of interest. Three components have to be delivered into the host cell line either by transfection or viral infection: vector AAV DNA containing the transgene of interest, Rep and Cap genes (also known as packaging construct), and helper genes from adenovirus. Rep78 and 68 promote AAV DNA rescue and subsequent replication. Cap proteins are synthesized in the cytoplasm and are shuttled to the nucleus for assembly. AAP supports assembly and maturation of the AAV capsid (Samulski and Muzyczka 2014). Rep52 and 40 interact with single-stranded DNA and pre-formed capsids to promote viral DNA encapsidation by a mechanism not yet fully understood (Ling et al. 2015). P, promoter; pA, polyadenylation sequence

AAV is a non-enveloped, single-stranded DNA virus that belongs to the family *Parvoviridae*. The 4.7-kb genome contains two main open reading frames, Rep and Cap (Fig. 1a). Rep encodes four regulatory proteins (Rep78, Rep68, Rep52, and Rep40) that play important roles in replication and encapsidation of viral DNA. Cap encodes three capsid proteins (VP1, VP2, and VP3) and assembly-activating protein (AAP) that promotes capsid formation. The genome is flanked by inverted terminal repeats (ITRs) which contain Rep recognition sequences important for AAV DNA replication and packaging (Samulski and Muzycska 2014). As a *Dependoparvovirus*, AAV requires the aid of another virus to propagate in tissue culture. Adenovirus and herpes simplex virus have been traditionally used as AAV helper virus.

An AAV vector is a recombinant variant of the wild-type (wt) AAV virus, in which the natural coding and non-coding regions have been replaced by an expression cassette not bigger than 4.7 kb. The vector genetic construct retains the lateral ITRs which are the only cis-acting elements required for replication and encapsidation of AAV DNA. With these modifications, rAAV becomes a replication-deficient entity, capable only of infecting cells and delivering DNA into their nuclei. As shown in Fig. 1b, vector production in culture requires Rep and Cap genes to be provided in a separate construct (Kotterman and Schaffer 2014). AAV genetic elements can be re-arranged in multiple constructs and then delivered into host cells via plasmid transfection or viral infection. Helper virus activities are also required for efficient vector production. They are provided by coinfection with helper virus stock or transfection with a plasmid containing “helper” genes.

## Molecular design of rAAV-producing expression systems

### Adenovirus complementation system

Early AAV-producing systems contained three components: a plasmid with the transgene of interest flanked by ITRs, Rep and Cap genes expressed from wild-type AAV or from a second plasmid, and AdV to provide helper functions (Tratschin et al. 1985). These systems showed positive AAV-like biological functionality (i.e., AAV DNA replication, formation of AAV full particles, DNA rescue, and replication after AdV coinfection) and served as a proof of concept for rAAV production. However, this process had limited potential for clinical use due to low yield and the presence of process-related impurities such as contaminating AdV and replication-competent (rc) AAV.

**Fig. 2:**
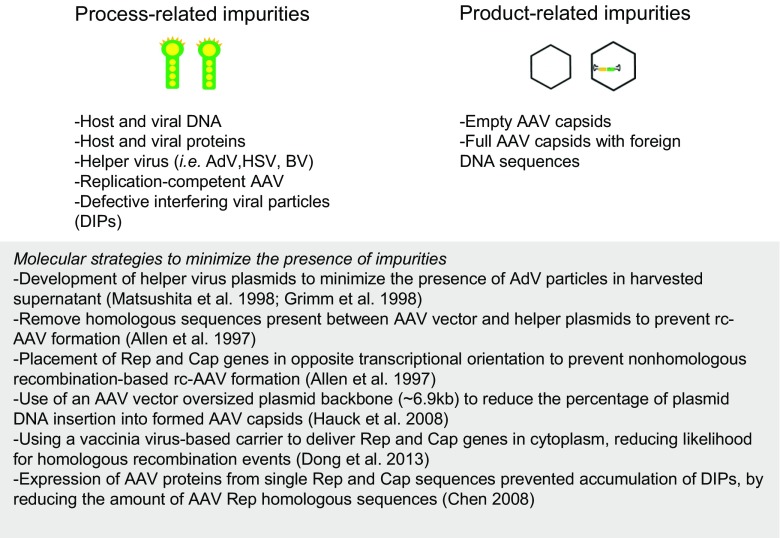
rAAV production-related impurities and molecular strategies aimed for their reduction. Adapted from Wright (2014)

Subsequent approaches based exclusively on plasmid transfection removed the need for wild-type AdV coinfection and usually require transfection with two or three plasmids containing the vector sequence (ITR-transgene-ITR), Rep/Cap genes, and helper virus auxiliary genes. Matsushita et al. (1998) evaluated different helper plasmid configurations in the search for the minimal set of genes required for AdV-free AAV vector production. Their best design included one plasmid with combined AAV vector and Rep/Cap genes and a second plasmid containing VA RNA, E4orf6, and E2A adenoviral genes. Because the HEK293 host cells already constitutively expressed the adenovirus proteins E1a and E1b, the full helper gene set included the five aforementioned proteins whose role on AAV production has been determined (Weitzman and Leiden 2011). This configuration tackled the initial problem of formation of replication-competent AAV and removed the need for infectious adenovirus. In parallel, reduction of rc-AAV formation was also accomplished by altering homologous sequences present in both vector and helper plasmids (Allen et al. 1997). Subsequent efforts aimed at reducing process and product-related impurities have been reported (Fig. 2).

Despite design improvements, per cell productivity was lower than wild-type AAV viral yields (Clark 2002; Samulski and Muzyczka 1999). Several observations on natural AAV production indicated that the higher particle per cell yields might be linked to (1) higher Rep and Cap gene copies per cell because of ITR-based DNA self-replication and (2) controlled expression of Rep78 levels has a positive impact on vector yield, and ameliorate Rep-mediated cytotoxicity which otherwise would impact cell viability (Schmidt et al. 2000; Xiao et al. 1998). Based on those premises, several groups proposed variations of the initial design. Xiao et al. (1998) used a two-plasmid system for enhanced vector production. The packaging plasmid, pXX2, contained an unconventional initial codon ACG on the Rep gene to modulate its expression. In addition, this plasmid conformation included two p5 promoters (upstream of Rep and downstream of Cap) to improve p5’ enhancer-like activity on the p40 promoter. The final yields obtained reached 10^5^ particles per cell, which exceeded the values obtained from Matsushita by nearly tenfold. Grimm et al. (1998) developed a pDG packaging plasmid, which combined Rep and Cap genes with AdV E2A, E4, and VA RNA helper genes. AAV p5 promoter was replaced by an MMTV-LTR promoter, which correlated to a reduction in Rep78 and increase in VP levels. Other molecular configurations were proposed by different groups, obtaining comparable vector yields (Allen et al. 2000; Collaco et al. 1999; Li and Samulski 2005; Table 1).

**Table 1:** Reported rAAV vector yields on the articles cited in this mini-review

	Method/design	Vector yield	Reference
AdV-based system			
	Ad-free, triple plasmid transfection	120ETU/cell	Matsushita et al. (1998)
	Transient transfection, pXX2, unconventional start codon to modulate Rep expression	1.2 × 10^3^ETU/cell 9.4 × 10^5^ vg/cell	Xiao et al. (1998)
	Rep/Cap Hela stable cell line + AdV	up to 36 IP/cell	Clark et al. (1995)
	Transient transfection, pSH3/pSH5 plasmids which combined AAV Rep, Cap, and AdV-helper genes	1.3 × 10^4^ vg/cell 135 ETU/cell	Collaco et al. (1999)
	Transient transfection with pDG plasmid, MMLV regulate Rep expression	150 IP/cell	Grimm et al. (1998)
	Transient transfection, Mtrep-CMVcap plasmid, E4orf6-only plasmid	10^4^ vg/cell 23 TU/cell	Allen et al. (2000)
	Rep/Cap stable cell line A549 + AdV	262 TU/cell	Gao et al. (2002)
	Rep/Cap stable cell line + AdV-Cre vector	1.3 × 10^5^ vg/cell 1.7 × 10^3^ ETU/cell^a^	Qiao et al. (2002)
	Self-replicating Rep/Cap helper construct	2 × 10^9^ IU/well	Li and Samulski (2005)
	Transient transfection, Rep/Cap split system	2.6 × 10^5^ vg/cell 37.8 TU/cell	Emmerling et al. (2016)
HSV-based system			
	Transient transfection, HSV-rc/d27 amplicon system	480 vg/cell	Conway et al. (1997)
	Infection with rHSV-rc strain on AAV-GFP-integrated cell line	480 ETU/cell	Conway et al. (1999)
	Infection with rHSV Rep/Cap and rHSV-GFP	1.5 × 10^5^ vg/cell 6 × 10^3^IP/cell	Hwang et al. (2003)
	Double infection with ICP27-deleted rHSV strains	40 TU/cell	Booth et al. (2004)
	Production of rAAV serotypes 1,2, and 9, by double infection with ICP27-deleted rHSV strains	> 1.3 × 10^5^ vg/cell > 9 × 10^3^ IP/cell	Kang et al. (2009)
	Production of rAAV serotypes 1,2,5 and 8, by using suspension-adapted BHK cells infected with rHSV strains	up to 1 × 10^5^ vg/cell up to 1 × 10^4^ IP/cell	Thomas et al. (2009)
	Suspension-adapted BHK cells infected with rHSV strains	> 5 × 10^4^ vg/cell	Knop et al. (2011)
BV-based system			
	Initial design, triple-BV system, Rep/Cap genes controlled by IE1/*polh* promoters	5 × 10^4^ vg/cell 30 TU/cell	Urabe et al. (2002)
	Swapping of AAV genetic elements from different serotypes to improve production of rAAV5	6 × 10^4^ vg/cell	Urabe et al. (2006)
	Intron-splicing mediated expression	1 × 10^11^ vg/mL	Chen (2008)
	Stable cell line, integration of Rep/Cap sequences + hr2-0.9 homologous regions + RBE sites	> 10^5^ vg/cell	Aslanidi et al. (2009)
	Stable cell line producing rAAV serotypes 1–12	up to 5 × 10^5^ vg/cell	Mietzsch et al. (2014)
Yeast expression system			
	4-plasmid system, individual expression cassettes regulated by Gal1/Gal10 promoters	~ 10^8^ vg/mL	Barajas et al. (2017)

*vg* vector genomes, *ETU* enhanced transducing unit, *TU* transducing unit, *IP* infectious particle, *IU* infectious unit

^a^Assuming 5 × 10^6^ cells per 10 cm plate were used for assay

**Fig. 3:**
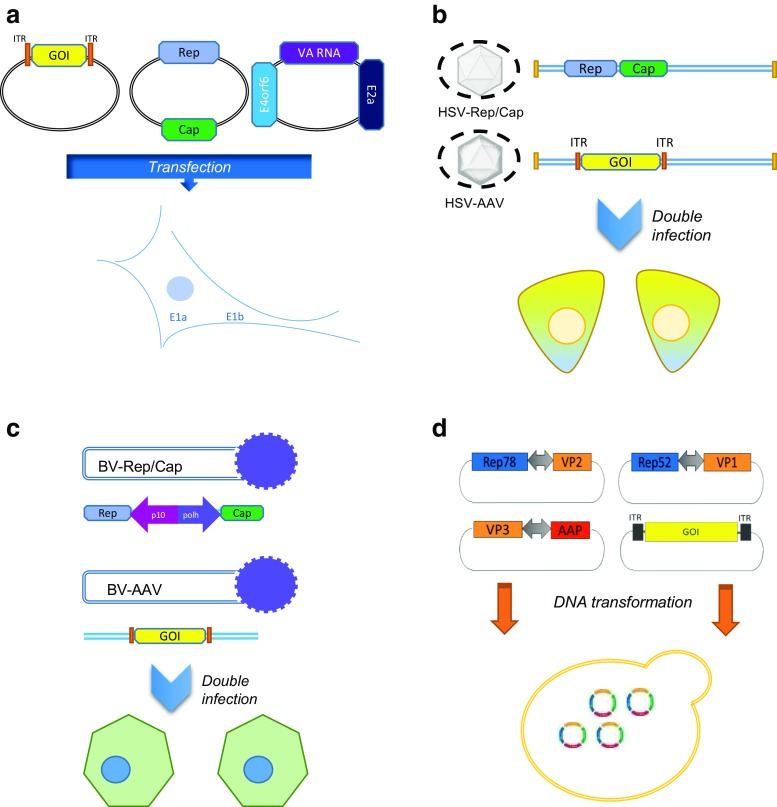
rAAV-producing systems: Production in adenovirus complementation systems (**a**) are traditionally performed as plasmid transfection processes, where AAV Rep/Cap genes, the ITR-flanked gene of interest (GOI), as well as AdV-helper genes are provided to a E1a/E1b-containing HEK293 cell line. HSV complementation systems (**b**) use two recombinant herpes viral strains to provide AAV Rep/Cap genes, GOI, and HSV-helper elements to a mammalian cell line such as BHK. Sf9—baculovirus expression systems (**c**) require two recombinant BV viral strains to provide the AAV-producing capability to insect cells. AAV protein expression is controlled by Sf9 natural promoters. Yeast-based systems (**d**) are transformed with a set of extrachromosomal plasmids that contain six AAV expression cassettes and GOI. AAV protein expression is controlled by yeast natural promoters

Such research efforts led to the adoption of the triple plasmid transfection system as one of the preferred methods for rAAV production. The standard configuration of this system provides the original Rep and Cap genes with their natural promoters (p5, p19, and p40), but striped from other cis-acting elements to reduce the probability of encapsidation. Under this configuration, one plasmid contains Rep and Cap genes, a second plasmid comprises the ITR-flanked transgene of interest, and a third plasmid provides helper genes (Fig. 3a). Recently, Emmerling et al. (2016) evaluated a new Rep/Cap split packaging plasmid system, in which the original Rep and Cap genes were segregated into two plasmids. In the first plasmid, the Rep gene was split into two expression cassettes, one for Rep68 and another for Rep52 and Rep40. A second plasmid contained the Cap expression cassette for expression of the three VP proteins and AAP from the natural p40 promoter. This study indicated that high yield correlated to an optimal Rep68/Rep52 expression ratio and enhanced Cap expression. Overall, a 12-fold increase in vector productivity (vg/cell) and a fivefold increase in transducing units/cell for this optimized plasmid transfection system were obtained, compared to the widely used pDG packaging system.

Although plasmid transfection methods offer simplicity and flexibility for basic research and early-stage rAAV production, these protocols have limited scalability and reproducibility for use in large-scale production. Alternatively, stable packaging and producer cell lines were designed. These two different systems contain Rep/Cap only or Rep/Cap plus vector constructs integrated into the cell genome, respectively. They all require the addition of Ad helper genes via plasmid transfection or virus infection, the latter being preferred to generate high yields (Chadeuf et al. 2000). This methodology improves cell culture process scalability and reduces the number of plasmids/virus required (Van der Loo and Wright 2016). Many variations of these stable cell lines have been designed (Gao et al. 1998; Gao et al. 2002; Clark et al. 1995; Qiao et al. 2002) giving vector genome titers ranging around 10^4^–10^6^ vg/cell.

### Herpesvirus complementation system

Early studies performed by Weindler and Heilbronn (1991) confirmed that HSV coinfection allowed AAV propagation and that genes UL5/8/52 and DNA-binding protein gene UL29 were responsible for helper-like activities. The function of these HSV helper genes has also been determined (Weitzman and Leiden 2011). The HSV system became an alternative platform that could overcome production challenges identified in AdV-based systems, namely the complexity of large-scale transfection methods, as well as the presence of helper virus impurities. Early HSV plasmid designs used an HSV1 amplicon expressing AAV2 Rep and Cap proteins, plus wild-type HSV and a vector construct (Conway et al. 1997). This initial design was later improved by developing an ICP-27-deficient HSV strain which expressed AAV Rep/Cap (Conway et al. 1999). This recombinant strain was incapable of replicating in culture, reducing the generation of impurities throughout the process. Replication-deficient rHSV Rep/Cap propagation was performed as a separate process, by cultivating the virus in ICP27-expressing Vero cells. Efficient production of rAAV on HEK293 cells was accomplished by transducing cells with rHSV Rep/Cap and transfecting an AAV-GFP vector plasmid. Slightly better yields were observed when an HEK-derived, proviral cell line (GFP-92) was used. The use of replication-deficient rHSV shows no detectable levels of rc-AAV and low levels of viral helper particles in culture (Clement et al. 2009).

Current HSV-based design comprises two replication-deficient HSV strains engineered to individually harbor Rep/Cap and AAV vector sequences (Fig. 3b). This transfection-free approach was initially reported by Hwang et al. (2003), who were able to improve vector yield 30-fold relative to a transfection-based method after optimizing HSV multiplicity of infection (MOI) ratios (12 and 2 for rHSV/RepCap and rHSV/AAV-GFP viruses, respectively). Kang et al. (2009) expanded this rHSV-based, AAV production platform across multiple serotypes and transgenes with proven efficiency (higher than 1 × 10^5^ particles/cell). Other groups adapted the infection process to suspension culture. Per cell productivity obtained after infecting suspension-adapted BHK cells ranged around 8 × 10^4^–2 × 10^5^ vg/cell (Thomas et al. 2009; Knop et al. 2011). This modification allowed easier scale up and generated high yields. Current efforts are focused on improving viral inoculum growth and stability in culture.

### Insect cell—baculovirus expression system

The baculovirus (BV) complementation system has become a reliable platform for expression of single heterologous proteins and multimeric particles. Using this method, recombinant viral strains usually derived from *Autographa californica* multinuclear polyhedrosis virus (AcMNPV) infect insect cells, hijacking the cellular machinery and expressing proteins encoded in the genome, including the foreign protein of interest. High per cell productivity yields are achieved because of BV’s strong promoters. Insect cell lines such as Sf9 or Sf21 can grow in suspension and are easily adapted to scalable, stirred tank bioreactor-based processes (Merten et al. 2005). Evidence of human-like post-translational modification capacity and intracellular viral capsid assembly support its use as a production platform for complex surface antigens, virus-like particles (VLPs), and potentially fully assembled viral vectors (Van Oers et al. 2015; Fernandes et al. 2013).

Under these premises, several groups developed BV constructs that promoted AAV capsid formation, DNA replication, and subsequent packaging within Sf9 cells. Urabe et al. (2002) designed an rAAV2 production strategy based on infection of Sf9 cells with three different recombinant BVs. The first BV carried Rep78 and Rep52 genes, the second carried the Cap gene, and the third carried the transgene flanked by ITRs. Preliminary experiments showed impaired Rep expression when AAV natural promoters were used; therefore, BV-specific promoters were adopted. Rep 78 and Rep52 expressions were expressed from two independent cassettes controlled by baculovirus immediate early (ΔIE1) and polyhedrin (*polh*) promoters, respectively. The three VP proteins were expressed from a single cassette controlled by the *polh* promoter, and the VP1 start codon was mutated to ACG to enable expression of the three VP proteins at the appropriate ratio, without the need of alternative splicing. Per cell productivity with this system achieved 5 × 10^4^ vg/cell. The final product resembled HEK293-produced AAV vectors in its physical and biological properties. The same author later developed a BV infection process for production of rAAV5 that achieved considerable yield (~ fourfold) and infectivity improvements by swapping in serotype 1 Rep52 for the serotype 5 analog and the N-terminal portion of serotype 2 VP1 for the analogous portion from serotype 5 (Urabe et al. 2006). Chen (2008) further modified the system to allow expression of multiple proteins from single Rep and Cap coding sequences. *Polh* promoters were placed within intron sequences to drive expression of Rep and Cap short transcripts, allowing the production of Rep52, VP2, AAP, and VP3 proteins. Rep78 and VP1 proteins were translated from longer transcripts after splicing of the internal *polh* promoter sequence. Under this concept, Rep78/52 and VP1/2/3 expression was accomplished by infection with either one or two BVs. Yields of 10^14^ vg/L were reached with the two-BV and three-BV proposed systems. Alternatively, Smith et al. (2009) were able to express Rep genes from a single mRNA species based on an mRNA leaky scanning mechanism, in which several AUG codons found in the Rep 78 initial sequence (including the start codon) were mutated to suboptimal triplets, thus allowing translation of Rep52 by the ribosome. This concept was applied for both Rep and Cap, and the generated sequences were cloned in opposite transcriptional orientation and consolidated into one Rep/Cap BV.

Overall, the new two-BV design (Fig. 3c) supported process robustness and scalable production of infectious rAAV by reducing the number of required BV viruses and additionally increased virus stability. The latter aspect becomes relevant when using replication-competent baculovirus for vector production, as defective-interfering viral particles (DIPs) can emerge during culture. DIPs are variants that gain a competitive advantage against complete baculovirus by deleting subsets of genes. Outgrowth of DIPs correlates to loss of transgene and reduced rAAV productivity in insect cells. Strategies to prevent DIP formation during rAAV production include BV inoculum optimization at minimum multiplicity of infection (MOI) and molecular changes in the BV that reduce the number of homologous Rep sequences (Cecchini et al. 2008).

The generation of stable insect cell lines for production of recombinant AAV vectors has also been explored. Aslanidi et al. (2009) developed an inducible system that required infection with a single BV strain. The Rep and Cap sequences, controlled by baculovirus promoters, were integrated within the Sf9 genome, and subsequent AAV gene amplification was triggered as a result of infection with a BV carrying an ITR-flanked transgene. The addition of a Rep binding element (RBE) upstream of Rep and Cap likely promoted feed-forward amplification of AAV gene expression. Overall, vector yields were improved by tenfold in comparison to the three-BV process. Mietzsch et al. (2014) further expanded Aslanidi’s design and generated a production platform for rAAV serotypes 1–12 called OneBac. Rep genes from AAV2, 4, or 12 were used in combination with the Cap genes to generate yield improvements similar to Aslanidi (up to 5 × 10^5^ vg/cell), but now for all serotypes. Mietzsch, however, noticed a decrease in VP1 levels and vector infectivity for rAAV5. Further improvements of this Sf9-infected stable production system (Mietzsch et al. 2015) based on the intron-splicing approach proposed by Chen (2008) led to a recovery of rAAV5 infectivity. Additionally, the authors were able to reduce collateral packaging of baculovirus genomic DNA by removing the previously integrated RBE signal.

The insect cell BV expression system is one of the most promising platforms for recombinant AAV production. The Sf9 system’s per cell productivity and volumetric productivity are among the highest documented to date (> 10^5^ vg/cell; ~ 10^11^ vg/mL) (Samulski and Muzyczka 2014). Efforts are currently underway to further improve cell-specific productivity, BV stability, and process scalability.

### AAV-producing yeast system

*Saccharomyces cerevisiae* is a unicellular, eukaryotic organism commonly used in research and technology. Baker’s yeast’s features include simple growth requirements, well-understood genetics, and post-translational protein processing comparable to complex eukaryotic systems excepting subtle differences in N-linked glycosylation patterns (Nielsen 2013). These characteristics make *Saccharomyces* suitable not only as a model system for eukaryotic cell biology studies, but also as a heterologous protein expression system for biotechnology applications. *Saccharomyces*’ value has been explored in the pharmaceutical industry. Recombinant strains capable of producing therapeutic proteins, antigenic proteins, and virus-like particles (VLPs) have been generated. Its proven record of heterologous viral protein expression, evidence of assembled capsid production, and evidence of virus replication of some entities such as *Parvovirus* suggested the possibility of generating full AAV viral particles in this organism (Kim and Kim 2016; Bill 2015; Zhao and Frazer 2002a; Zhao and Frazer 2002b).

Recent studies investigated rAAV generation in yeast. Backovic et al. (2012) utilized various plasmid constructs to demonstrate capsid protein expression and AAV capsid assembly in *Saccharomyces*. Their initial design placed the Rep and Cap genes under the control of their natural promoters along with an intron placed upstream of the VP1 initiation sequence. This design allowed the recovery of VP3 protein only. A second design with the Cap gene under a Gal1 promoter and a Kozak region upstream of VP1 facilitated expression of VP1. Cotransformation with the previously mentioned plasmids and optimized gene induction led to successful expression of VP1 and VP3 at a ratio comparable to theory. Transmission electron microscopy studies confirmed capsid morphology of the purified product. Moreover, the same group demonstrated AAV single-stranded genome replication dependent on Rep expression and the presence of ITRs. DNA analysis of replicated sequences led the authors to suggest that AAV DNA replication process in yeast appear to be dissimilar from AAV canonical replication (Cervelli et al. 2011).

Barajas et al. (2017) demonstrated production of fully assembled, infectious AAV particles in *S*. *cerevisiae*. The system was based on four plasmids containing individual expression cassettes for two Rep proteins (Rep78 and Rep52), three VP capsid proteins, and the assembly-activating protein (AAP) (Fig. 3d). Unlike previous efforts, protein expression from the six expression cassettes was controlled by yeast-specific, galactose-inducible GAL promoters of different strengths, and codon optimization was required on Rep and AAP sequences. Southern blot analysis demonstrated formation of AAV DNA monomeric forms, and Western blot analysis of purified capsids showed detectable levels of the three VP capsids. Further examination confirmed transgene presence and infectious capacity of the purified material. The authors reported rAAV2 full particle titer yield that ranges around 10^8^ vg/mL. This four-plasmid system design aligns with other similarly proposed plasmid configurations (Thakur 2002; Snyder 2011), and altogether, these studies constitute the proof-of-concept of rAAV production in a microbial system. The reported results not only demonstrate the potential utility of the yeast system as a tool to investigate AAV biology; this new concept raises the possibility of potentially developing an alternative, cost-effective, highly scalable platform for rAAV production at large manufacturing scale. However, low vector yields and poor DNA encapsidation rates limit its application. Certain functionalities like those provided by helper viruses and host factors in other systems may be suboptimal in yeast and would need to be supplemented for high yield rAAV production. More investigations are needed to understand the benefits and limitations of this system that, although promising, is still far from becoming an efficient rAAV production platform.

## Final remarks

The molecular design of rAAV-producing expression systems plays a critical role not only in per cell vector productivity, but also in process robustness and elimination of process and product-related impurities. Each production system has advantages and disadvantages when adopted in pre-clinical or clinical manufacturing environments. AdV complementation systems based on triple plasmid transfection process are commonly adopted in lab settings because of their simple rAAV production workflow, flexibility to switch production to different AAV serotypes, and proven productivity and product quality. Scaling up this system, however, brings challenges associated to cell adaptation to suspension culture, plasmid generation, and lot-lo-lot variability in transfection efficiency. The HSV complementation system based on the use of rHSVs overcomes scale-up limitations regarding to cell culture scale-up and process variability, while maintaining high productivity at large scale. One of the main challenges of this system relies on the viral inoculum stability and propagation. Production of AAV vectors in the Sf9/BV complementation system has proven to be very efficacious, and several studies demonstrated the suitability of this system for large-scale vector production. However, like other systems based on viral infection, special attention needs to be focused on the viral inoculum stability and generation. Additionally, more studies need to be performed to fully characterize quality attributes of Sf9-based vector material (Wang et al. 2011).

All rAAV expression systems have three fundamental commonalities: (1) successful delivery and amplification of the necessary genetic material into the host cell line, (2) fine-tuning of Rep-Cap expression levels, and (3) modification of the cellular milieu to a more “AAV-friendly” environment—something that has only recently begun to be investigated. The use of non-native hosts for rAAV production brings also the need to optimize critical variables such as timing and strength of expression of AAV components.

Besides molecular optimization of AAV genes, some research has focused on identifying limitations imposed by the host cells on rAAV production or the negative effects caused by rAAV on the cells. Satkunanathan et al. (2014) identified Y-box binding protein (YB1) as an inhibitory protein of rAAV production. They postulated this protein could interfere with viral DNA encapsidation by competitive binding for recognition sequences. Experimental silencing of this gene translated into a significant increase in rAAV2 titer relative to control condition. Reid et al. (2017) hypothesized producer cells could experience cytotoxicity driven by rAAV transgene overexpression. mRNA silencing studies on different transgenes resulted in yield improvement up to 22-fold relative to control. Future research efforts in multiple directions will hopefully translate to highly productive vector expression systems.
